# Utilizing patient-specific 3D printed guides for graft reconstruction in thoracoabdominal aortic repair

**DOI:** 10.1038/s41598-021-97541-8

**Published:** 2021-09-09

**Authors:** Taehun Kim, Dayeong Hong, Junhyeok Ock, Sung Jun Park, Younju Rhee, Sangwook Lee, Guk Bae Kim, Dong Hyun Yang, Joon Bum Kim, Namkug Kim

**Affiliations:** 1 Department of Convergence Medicine, Asan Medical Institute of Convergence Science and Technology, Asan Medical Center, University of Ulsan College of Medicine, Seoul, Republic of Korea; 2 Department of Biomedical Engineering, Asan Medical Institute of Convergence Science and Technology, Asan Medical Center, University of Ulsan College of Medicine, Seoul, Republic of Korea; 3 Department of Radiology, University of Ulsan College of Medicine, Asan Medical Center, Seoul, Republic of Korea; 4 Department of Thoracic and Cardiovascular Surgery, Asan Medical Center, University of Ulsan College of Medicine, Seoul, Republic of Korea; 5 ANYMEDI Inc., 388-1 Pungnap2-dong, Songpa-gu, Seoul, South Korea

**Keywords:** Biomedical engineering, Clinical trial design

## Abstract

In thoracoabdominal aortic aneurysm repair, repairing the visceral and segmental arteries is challenging. Although there is a pre-hand-sewn and multi-branched graft based on the conventional image-based technique, it has shortcomings in precisely positioning and directing the visceral and segmental arteries. Here, we introduce two new reconstruction techniques using patient-specific 3D-printed graft reconstruction guides: (1) model-based technique that presents the projected aortic graft, visualizing the main aortic body and its major branches and (2) guide-based technique in which the branching vessels in the visualization model are replaced by marking points identifiable by tactile sense. We demonstrate the effectiveness by evaluating conventional and new techniques based on accuracy, marking time requirement, reproducibility, and results of survey to surgeons on the perceived efficiency and efficacy. The graft reconstruction guides cover the segmentation, design, fabrication, post-processing, and clinical application of open surgical repair of thoracoabdominal aneurysm, and proved to be efficient for accurately reconstructing customized grafts.

## Introduction

3D printing (3DP) technology is used in the medical field for patient-specified guides, simulators, surgical planning, education, and implants^1–7^. 3DP technologies, rapid prototyping and additive manufacturing, proceed by adding materials layer-by-layer until the object is completely built^3,8^. In addition to reducing the traditional processes, time, and cost, it also enables the fabrication of complex structures. In addition, using various materials, 3DP makes it possible to realise a virtual 3D model as a physical phantom, unlike existing technologies^1,3,6,9–11^.

Aortic aneurysms are life-threatening disorders that increase the risks of aortic dissection or rupture with fatal outcomes^12^. Surgical or interventional therapies that anatomically replace the diseased aortic segments are the only treatments proven to prevent catastrophic aortic events. Within this disease entity, thoracoabdominal aortic aneurysm is the most extensive and challenging pathology; it requires the replacement of the diseased aorta including the downstream thoracic and abdominal segments, along with the reconstruction of the visceral arteries (celiac and superior mesenteric artery), bilateral renal arteries and several segmental arteries (intercostal and lumbar arteries) that supply blood to the spinal cord^13^. To perform this extensive surgical procedure, extracorporeal circulation, as well as selective perfusion to visceral and renal arteries are mandatory, both of which increase the invasiveness and level of surgical stress. Open surgical repair of thoracoabdominal aorta is well known as the most difficult and challenging surgical procedure, and operative outcomes remain reportedly poor, even by the world-leading expert groups, and the rates of surgical mortality, major stroke, and paraplegia are 9.5%, 11.6%, and 13.9%, respectively^13^.

Various efforts have been made to reduce such related risks in thoracoabdominal aorta surgery, including the use of the four-branched aortic graft for more efficient revascularizations of aortic branching arteries through the aid of commercially available products. In addition to this technique, the use of an eight-branched aortic graft—so called “octopod graft technique,” has also been introduced to enable efficient revascularization of the spinal cord feeding arteries, which are known to be pivotal in preventing paraplegia^14^. The octopod technique involves constructing a pre-sewn multi-branched aortic graft before surgery through an image-based technique (IBT); it entails grafting branches for the intercostal and lumbar arteries (spinal cord suppliers) on commercially available four-branched grafts considering the anatomical relationship based on the preoperative computed tomography (CT) images because the anatomical location of these vessels significantly vary among patients^15–18^.

However, this conventional image-based approach has several shortcomings with respect to the precise positioning of the branching grafts because the accuracy depends on the constructor, usually, the operating surgeon. To overcome this limitation, 3DP-based reconstruction of the thoracoabdominal graft has been introduced to enable more accurate construction of the aorta and branching vessels^4^. As an extension of this approach, we developed two reconstruction techniques using patient-specific 3D printed graft reconstruction guides: (1) model-based technique (MBT), which uses a visualization model designed as a realistic-shaped graft model that contains the main aortic body and its branching vessels, making it possible to manually position the branching grafts on the artificial aortic graft and (2) guide-based technique (GBT) using the marking guides, wherein the branching vessels in the visualization model are substituted with slightly protruded marking points. We evaluated the accuracy and marking time efficiency of three techniques (conventional IBT, MBT, and GBT) in terms of the proper positioning of the aortic branching vessels, using a designed graft model (DGM) as the gold standard.

## Methods

This retrospective study was carried out in accordance with the principles of Declaration of Helsinki and current scientific guidelines. The institutional review board for human investigations at Asan Medical Center approved this study with a waiver of informed consent from patients because of the use of retrospective clinical and imaging data. The data were de-identified, in accordance with the Health Insurance Portability and Accountability Act privacy rule. All methods were performed in accordance with the relevant guidelines and regulations.

**Figure 1:**
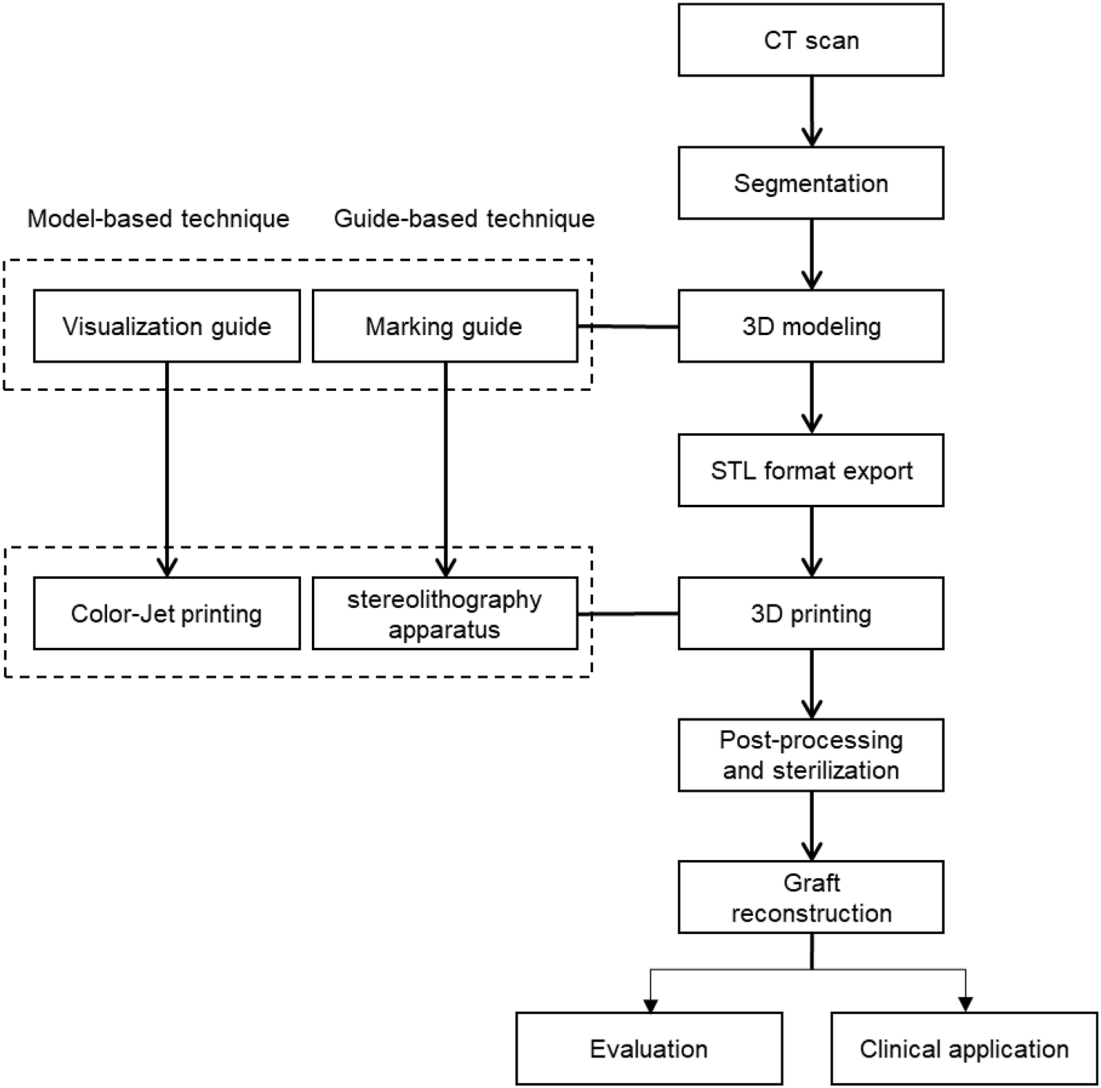
Overall process of two types of graft guide with MBT and GBT.

The procedure for two types of graft guide techniques, MBT and GBT, are illustrated in Fig. 1. The visualization model and marking guides segmented based on CT images were designed, modelled, and exported into the standard tessellation language (STL) format. Then, they were printed using ColorJet printing (CJP) and the stereolithography apparatus (SLA) technology, following which they underwent post-processing and ethylene oxide gas sterilization. Graft reconstruction was performed using the MBT and GBT for clinical application and the accuracy and time requirements were evaluated.

### Dataset acquisition

The imaging and clinical data of 15 patients diagnosed with Crawford extent II or III thoracoabdominal aortic aneurysm were used. These patients underwent open surgical aortic repair using 3DP in Asan Medical Center between Jan. 2017 and Feb. 2020. The segmental arteries to be reconstructed were selected from among those located between the T8 and L2 levels, and the main stem aortic graft reconstruction ranged from the left subclavian artery to infra-renal abdominal aorta or to the bilateral common iliac artery. The individual profiles of the subjects are detailed in Table 1. All the subjects underwent CT angiography (Siemens SOMATOM series, Siemens Healthcare) with 0.6–3 mm slice thickness and 70–120 kVp.

**Table 1:** Individual profiles of the subject patients including classification, level of segmental artery, and range of replacement.

Patients no.	Age	Sex	Operation date	Classification (extent)	Level of segmental arteries			Range of graft reconstruction
					Side	Thoracic	Lumbar	
1	36	W	12 Jan. 2017	II	Rs	10	–	LSA to IMA
					Ls	9, 10		
2	57	W	17 Oct. 2017	II	Rs	8, 10	–	LSA to BIA
					Ls	12		
3	48	M	16 May. 2018	II	Rs	9, 10, 11, 12	–	LSA to RA
					Ls			
4	40	M	05 Jun. 2018	I	Rs	9, 11	–	LSA to IMA
					Ls	9, 10		
5	37	M	19 Jun. 2018	II	Rs	11, 12	–	T-9 to BIA
					Ls	11		
6	46	M	19 Jul. 2018	III	Rs	11, 12	–	LSA to BIA
					Ls			
7	33	M	06 Sep. 2018	II	Rs	8, 9	–	LSA to IMA
					Ls			
8	22	M	04 Jul. 2019	II	Rs	10, 11, 12	–	LSA to RA
					Ls			
9	65	M	27 Jun. 2019	III	Rs	10	–	T- 10 to BIA
					Ls	11		
10	51	W	07 Nov. 2019	III	Rs	10, 11	1	LSA to BIA
					Ls	10	1	
11	69	M	14 Nov. 2019	II	Rs	8, 11, 12	–	LSA to BIA
					Ls	11		
12	57	M	22 Nov. 2019	IV	Rs	10, 12	–	LSA to CA
					Ls	9, 10, 12		
13	22	M	19 Dec. 2019	II	Rs	9, 10, 12	1, 4	T- 9 to BIA
					Ls	11, 12	1	
14	38	M	09 Jan. 2020	III	Rs	7, 8, 10, 11, 12	1, 3	T-7 to BIA
					Ls	7, 8, 9, 10, 11, 12	7, 8, 9, 10, 11, 12	
15	40	M	23 Jan. 2020	II	Rs	8, 10, 12	2, 3, 4	LSA to BIA
					Ls	8, 11	3, 4	

*Rs* right side, *Ls* left side, *T* thoracic, *LSA* left subclavian artery, *IMA* interior mesenteric artery, *BIA* bilateral common iliac artery, *RA* renal artery.

### Conventional image-based technique

The procedure of the conventional IBT proposed by Park et al.^14^ is as follows: (1) In the axial view of the CT images, the position of the visceral and segmental (intercostal and lumbar) arteries are identified. (2) To evaluate the angle between the visceral and segmental arteries, a horizontal line and a virtual diverging line of the branch from the central spot of aorta are drawn on each axial slices of a CT image at the visceral and segmental levels, and the angles between both lines are measured at each level. (3) Based on the visceral artery, the height of the segmental artery is determined and the corresponding point is marked with measured angle on the main aortic graft. (4) These processes are repeated as many times as the number of segmental arteries to be reconstructed.

### Development of visualization model and marking guide

**Figure 2:**
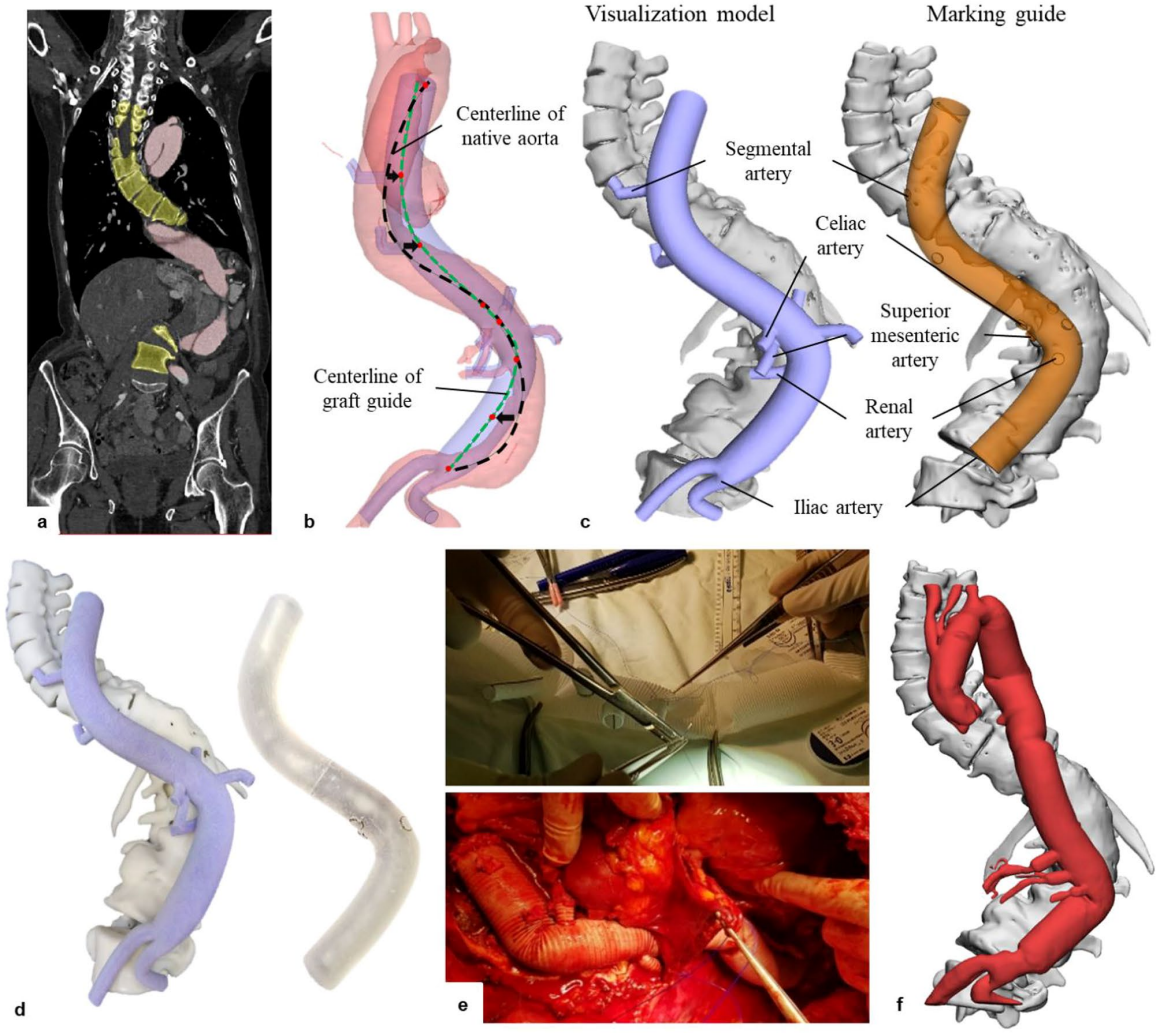
Two types of patient-specific graft reconstruction guide application for open repair of thoracoabdominal aortic aneurysm. (**a**) CT angiography images and segmentation of diseased aorta and spine; (**b**) modelling the centreline of graft based on that of a native aorta; (**c**) 3D modelling of the visualization model and marking guide; (**d**) 3D-printed visualization model and marking guide; (**e**) graft reconstruction and clinical application in operating room; (**f**) postoperative graft.

In manually segmenting the native aorta and spine, the major aortic branching vessels—the celiac artery, superior mesenteric artery, renal artery, bilateral common iliac artery, segmental arteries— using Mimics Medical 17 and 3-matic 9.0 software (Materialise Inc, Belgium) (Fig. 2a) were included. After 3D rendering based on the segmentation, the centreline of the native aorta was extracted through 3D computer-aided design (CAD) modelling and the squiggly centreline was modified to reflect the spine shape (Fig. 2b). The virtual model of the aorta was designed in the diameter of the prospective aortic graft to be implanted (24–30 mm); each of the virtual side-branches were united in 8 mm diameter as a single model. To achieve more efficient and accurate construction of the aortic graft, the graft reconstruction model and guide, visualization model, and marking guide, were developed: (1) The visualization model was designed as a realistic-shaped graft model containing the main aortic body and its branching vessels, to enable manual positioning of the branching grafts on the artificial aortic graft (Fig. 2c-left); (2) In the marking guide, the branching vessels in the visualization model were replaced by slightly protruding marking points (2 mm), to ensure that their positions were designed such as to be identifiable by tactile sense when wrapped by the aortic graft, instead of depending solely on visual mimicking (Fig. 2c-right). The DGM was validated by operating surgeons, following which it was exported as STL files.

### 3D printing technology

The visualization model was fabricated using a ColorJet printer (CJP) with VisiJet PXL Core powder, VisiJet PXL clear binder, and colour bonding materials (Fig. 2d-left). The marking guide was printed using a stereolithography apparatus (SLA) with Dental LT resin, a material capable of temporary mucosal contact (Fig. 2d-right). To fabricate the graft reconstruction guides, the layer thickness, field of view and X–Y plane resolution for the CJP were 0.10, 254 × 381 × 203 and 300 × 450 DPI, respectively, and 0.10, 145 × 145 × 145, 0.15 mm, respectively, for the SLA. The X–Y resolution of SLA was not precisely defined; therefore, Formlabs Inc. experimented the specimens to find the X–Y resolution^19^. In addition, the spot size and power of laser were 140 μm and 250 mW.

### Graft reconstruction method using MBT and GBT

**Figure 3:**
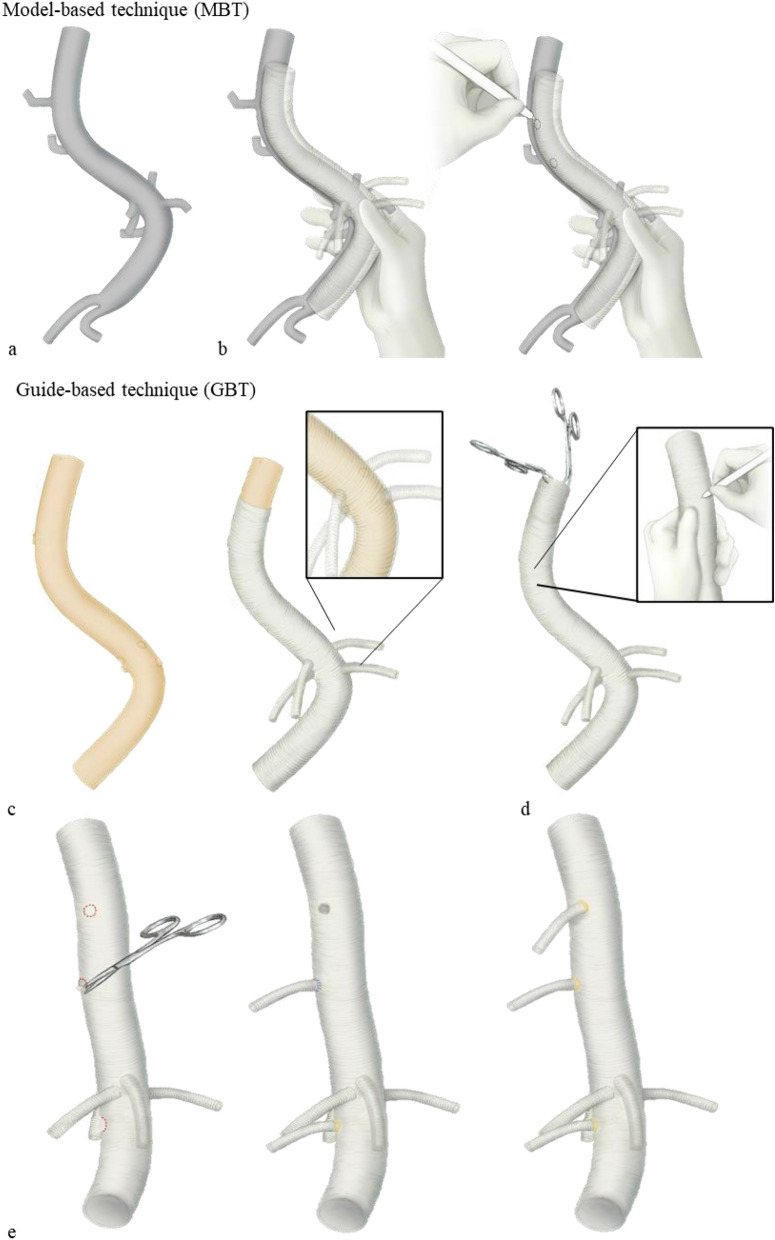
Patient-specific graft reconstruction process with MBT and GBT in operating room.

In a real operative setting, the grafts were pre-sewn by the operating surgeon with the patient during anaesthetic induction using either the MBT or GBT, and were used to surgically replace the diseased thoracoabdominal aorta (Fig. 2e). The postoperative configuration of the replaced aorta was confirmed by CT images (Fig. 2f). The experimental processes for patient-specific graft reconstruction using MBT and GBT are shown in Fig. 3. Under the supervision of the visualization model in the MBT, the shape of the main graft body was imitated (Fig. 3a) and the locations of the segmental vessels were indicated on the main graft body, with the point of the celiac artery origin as reference (Fig. 3b). In the GBT, the marking guide was inserted into the aortic graft and aligned with reference to the origin of the visceral arteries (Fig. 3c). Then, the positions of the segmental arteries were determined by the tactile sense through the protruding markers and marked on the graft surface (Fig. 3d). Both in the MBT and GBT, the segmental grafts were attached to the marked points by running sutures (Fig. 3e).

### Survey

To assess the convenience availed by the proposed techniques, three practicing cardiothoracic surgeons with experience in graft reconstruction with three techniques, IBT, MBT and GBT, were surveyed. The survey questionnaire was constructed in-house by curating aids to anatomical understanding, usefulness, satisfaction, surgical outcome, and recommendability from clinical standpoints. The surveyees had 3–10 years of experience as certified cardiothoracic surgeons. The questionnaire items were categorised as follows: (1) the level of contribution to understanding the anatomic structure (four questions); (2) usefulness for graft reconstruction (three questions); (3) satisfaction level for graft reconstruction (two questions); and (4) benefits of the use of 3DP technologies to surgical outcomes (two questions); and (5) recommendability of 3DP for thoracoabdominal aorta surgery to other surgeons (two questions) (Supplementary Table S1). The scores were recorded as 1 = Strong disagreement, 2 = Disagreement, 3 = Neutral, 4 = Agreement, and 5 = Strong agreement.

### Evaluation and statistical analysis

**Figure 4:**
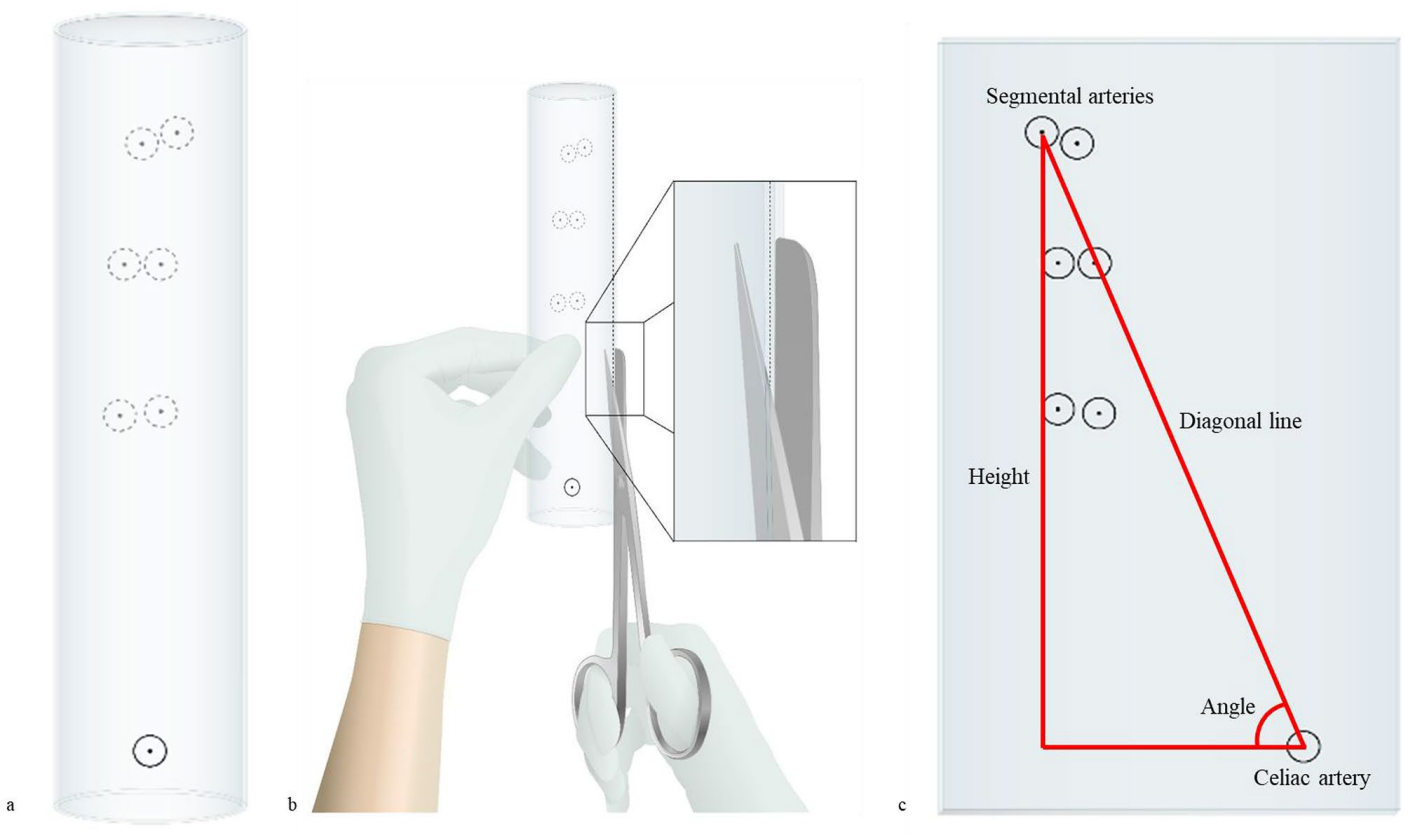
Guide to evaluating the accuracy of IBT and GBT; (**a**) Locating celiac artery and segmental arteries marked on graft. (**b**) Spreading the graft using scissors. (**c**) Measuring the length, height, and angle between celiac artery and segmental arteries.

The experiments of conventional IBT, MBT, and GBT performed by three researchers were measured, analyzed, and compared with the DGM based on the graft reconstruction accuracy and time required for fenestrations of vessels, excluding cutting out. This retrospective study was performed by applying three techniques to 15 patients, and mimic aortic grafts were fabricated to carry out the experiment. In the accuracy evaluation, the positions of the segmental arteries were indicated on the graft using the IBT, MBT and GBT (Fig. 4a), and the graft was unfolded using scissors (Fig. 4b). Then, the diagonal line, height, and angle between the designated celiac artery and the marked segmental arteries were measured (Fig. 4c).

The Bland–Altman analysis was used to compare the differences in the diagonal line, height, and angles of the DGM and three techniques, IBT, MBT and GBT, and to identify the LoA, bias, and outliers^20,21^. The inter-group differences in the measurements and marking time were evaluated using the paired t-test. The correlation coefficients (r) for the DGM and three techniques were observed, and based on the correlation analyses of the same sample size by an independent group, it was determined that the two correlations were statistically significant. An intraclass correlation coefficients (ICC) analysis was performed on the IBT, MBT and GBT experiments by three researchers to confirm the correlations among them. In addition, the survey results on the IBT and the MBT or GBT were analysed using the Wilcoxon signed rank test. All the statistical analysis was conducted using statistics software (SPSS 25.00, IBM; and Medcalc 19.6.4 free trial, Medcalc Software).

## Results

### Comparison of accuracy and marking time requirement of DGM and three techniques

**Figure 5:**
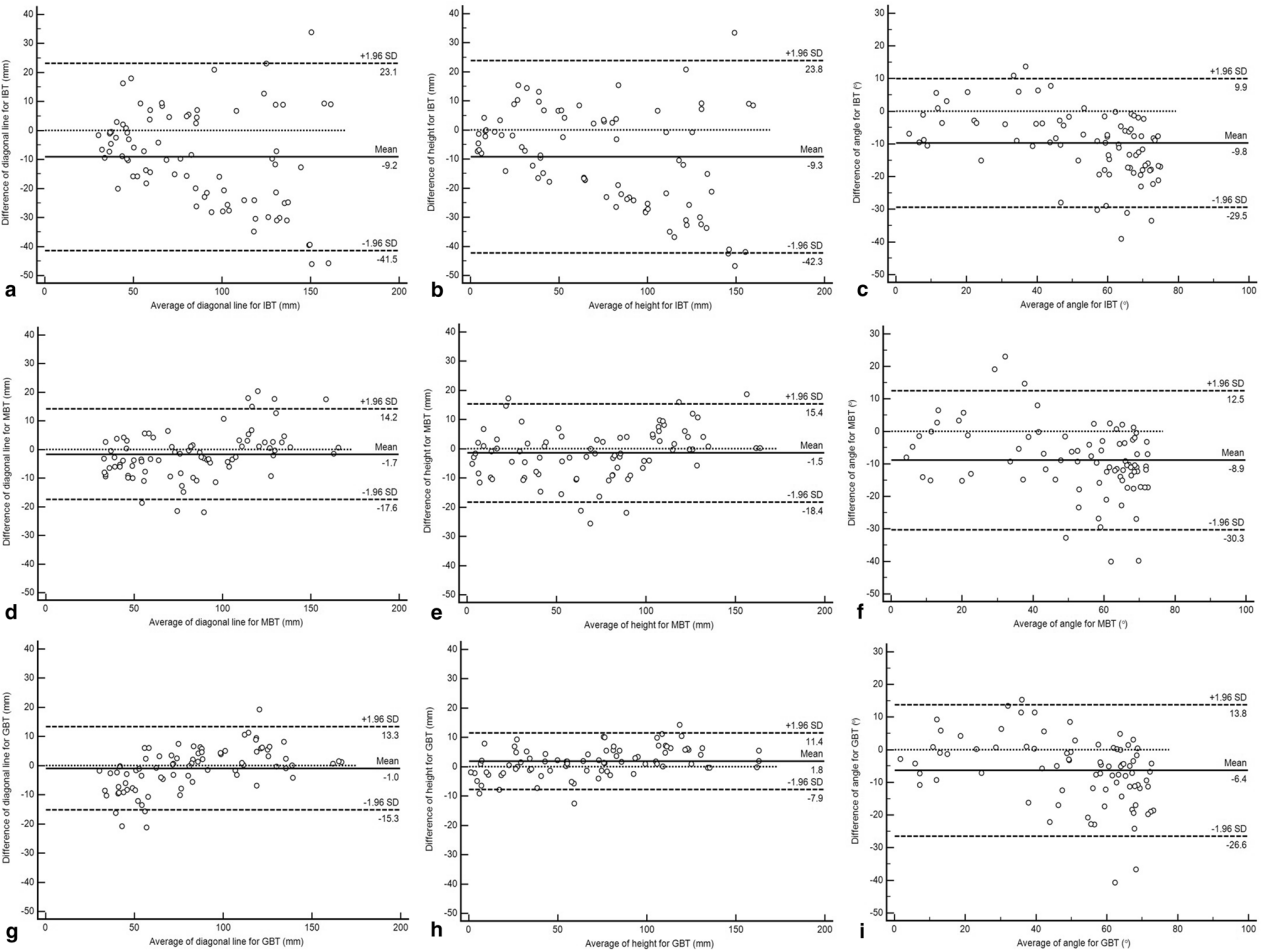
Bland–Altman plot indicating the distribution of the differences between DGM and IBT, divided by (**a**) diagonal line, (**b**) height and (**c**) angle; between the DGM and MBT, divided by (**d**) diagonal line, (**e**) height, (**f**) angle; and between the DGM and GBT, divided by (**g**) diagonal line, (**h**) height, and (**i**) angle.

Table 2 presents the measurement of the diagonal line, height, and angle from the celiac artery to the segmental arteries for the DGM, whose design was validated by surgeons, conventional IBT, MBT, and GBT. According to the Bland–Altman analysis, the arithmetic mean and standard deviation (SD) of the difference between the DGM and conventional IBT were − 9.19 ± 16.47 mm (limits of agreement (LoA): − 41.47 to 23.08 mm) for the diagonal line, − 9.25 ± 16.84 mm (LoA: − 42.26 to 23.76 mm) for the height, and − 9.76° ± 10.05° (LoA: − 29.47° to 9.95°) for the angle (Fig. 5a–c), respectively; the difference between the DGM and MBT was − 1.67 ± 8.11 mm (LoA: − 17.56 to 14.21 mm) for the diagonal line, − 1.50 ± 8.60 mm (LoA: − 18.35 to 15.36 mm) for the height, and − 8.89° ± 10.93° (LoA: − 30.31° to 12.52°) for the angle (Fig. 5d–f), respectively. The arithmetic difference values for the diagonal line, height, and angle between the DGM and GBT were − 1.00 ± 7.27 mm (LoA: − 15.25 to 13.24 mm), 1.79 ± 4.93 mm (LoA: − 7.86 to 11.45 mm), and − 6.39° ± 10.29° (LoA: − 26.55° to 13.78°) (Fig. 5g–i), respectively. There were statistically significant differences in all the measurements for the IBT, MBT, and GBT, except for the diagonal line of the GBT. The average marking time required for fenestrations of vessels excluding cutting out with three techniques are summarised in Table 2, and the time required for each of the 15 patients is shown in Supplementary Table S2. The MBT and GBT reduced the marking times by 17.6 and 15.5 min, respectively, compared with the conventional IBT.

**Table 2:** Measurements and time requirements of graft reconstruction using DGM, IBT, MBT, and GBT.

Measurements	DGM	IBT		MBT		GBT	
	Mean ± SD	Mean ± SD	p	Mean ± SD	p	Mean ± SD	p
diagonal line (mm)	82.19 ± 37.18	91.38 ± 42.38	< 0.001	83.87 ± 33.85	0.013	83.19 ± 33.27	0.053
Height (mm)	70.20 ± 43.99	79.46 ± 50.42	< 0.001	71.70 ± 41.77	0.028	68.41 ± 41.91	< 0.001
Angle (°)	47.38 ± 18.67	57.14 ± 23.21	< 0.001	56.28 ± 22.31	< 0.001	53.77 ± 22.52	< 0.001
Time	–	18.43 ± 9.67		0.80 s ± 0.25		2.90 ± 0.85	< 0.001*

*DGM* designed graft model, *IBT* image-based technique, *GBT* guide-based technique.

*There was a statistically significant difference between IBT and MBT, between IBT and GBT, and between MBT and GBT.

### Comparison of correlation among three techniques and researchers

The comparison results of the correlations coefficients of the DGM and three techniques, IBT, MBT, and GBT, based on their respective diagonal length, height, and angle are shown in Table 3. All the correlation coefficients exhibited strong correlation with statistical significances. The ICC of three independent researchers showed high levels of agreement with 0.986 for the conventional IBT and MBT, and 0.995 for GBT.

**Table 3:** Correlation coefficient between DGM and three techniques for diagonal length, height, and angle.

Comparing correlation coefficient	Diagonal line		Height		Angle	
	r_12_	p	r_13_	p	r_14_	p
**DGM**						
IBT	0.905	< 0.001	0.933	< 0.001	0.884	< 0.001
MBT	0.959	< 0.001	0.970	< 0.001	0.858	< 0.001
GBT	0.980	< 0.001	0.993	< 0.001	0.875	< 0.001

r_12_, correlation coefficient for DGM and IBT; r_13_, correlation coefficient for DGM and MBT; r_14_, correlation coefficient for DGM and GBT.

### Survey

The scores of the questionnaires are presented in Table 4 and Supplementary Table S1. The categories in the questionnaire were designed to rate graft reconstruction guides with the conventional IBT, MBT, and GBT on understanding, usefulness, satisfaction, surgical outcome, and recommendability for use by other surgeons for graft reconstruction in thoracoabdominal aortic repair. The average score for the MBT and GBT with the graft reconstruction guides was 59.33 ± 0.58 out of 65, whereas the conventional IBT had a relatively lower score of 20.67. For the contribution to understanding the anatomic structure, 58.3% of the answers indicated strong agreement, 25%, agreement, and 8.33%, neutral and disagreement for the MBT and GBT, whereas for the conventional IBT, 25% of the answers indicated neutral, 41.7%, disagreement, and 33.3%, strong disagreement. Under the usefulness for graft reconstruction, 88.9% of the answers indicated strong agreement and 11.11%, agreement, in the MBT and GBT, whereas 22.2% indicated disagreement and 77.8%, strong disagreement in the conventional IBT. Finally, for the MBT and GBT, satisfactoriness of graft reconstruction, surgical outcomes, and recommendability to other surgeons was 88.33%, 66.67%, and 16.67% of strong agreement, and 16.67%, 33.33%, and 83.33% of agreement, respectively, and, in the conventional IBT, 50% of disagreement and strong agreement were identified. Generally, the MBT and GBT scored higher than the conventional IBT with statistical significance.

**Table 4:** Retrospective survey in relation to understanding, usefulness, satisfaction, surgical outcome, and recommendability for use in other applications for IBT without 3D printing technique and MBT and GBT with 3D printing technique.

Classification	IBT	MBT and GBT	p
Understanding the anatomic structure	1.92 ± 0.79	4.33 ± 0.98	< 0.01
Usefulness for graft reconstruction	1.22 ± 0.44	4.88 ± 0.33	< 0.01
Satisfaction for graft reconstruction	1.50 ± 0.55	4.83 ± 0.41	< 0.05
Surgical outcomes	1.50 ± 0.55	4.67 ± 0.52	< 0.05
Recommendations for other applications	1.67 ± 0.52	4.17 ± 0.41	< 0.05

## Discussion

We developed two reconstruction techniques using patient-specific 3D printing graft reconstruction guides: (1) MBT using the visualization model designed based on the realistic-shaped graft model that contains the main aortic body and its branching vessels, making it possible to manually position the branching grafts on the artificial aortic graft and (2) GBT using the marking guides, in which the branching vessels in the visualization model were replaced by slightly protruding marking points. The patient-specific 3D printed graft reconstruction guides may provide following benefits: (1) the marking time can be minimized by checking and marking the ideal position of the patient’s segmental arteries in complex anatomy such as extremely tortuous spine and aorta. (2) With the improved accuracy and reproducibility of the aortic graft reconstruction, the techniques can be utilized easily even for those in the learning curves. (3) Owing to the improved procedural efficiency, the techniques may contribute to reducing the level of fatigue of operating surgeons during lengthy and exhausting surgery, thus increasing focus on important procedures.

Crawford extent II or III thoracoabdominal aortic aneurysm repair entails replacing the thoracoabdominal aorta and revascularising the visceral branches and intercostal or lumbar arteries simultaneously; therefore, it is regarded as the most extensive and challenging operation with high risks of surgery-related mortality and serious complications. The traditional Crawford’s island technique—a single aortic patch containing all four openings of visceral branches anastomosed with graft by side-to-side fashion—was previously accepted as a standard technique because it reduced the procedural burden^22^. However, it is reportedly common for patch aneurysm to develop in the remnant aortic island, especially among those with connective tissue disorders such as Marfan syndrome^23–27^. Although there are limited published reports on employing the pre-sewn multi-branched aortic graft in open repair of thoracoabdominal aortic aneurysm, its use is now a desirable option to prevent such aneurysm formation as observed in the conventional island-type aortic repair^25,27–29^. In addition, in cases where the ostia of the aortic branching arteries are displaced far away from each other, the use of a multi-branched graft may be the only way to effectively remove the diseased aorta.

Furthermore, paraplegia is a devastating complication of thoracoabdominal aortic aneurysm repair, particularly in extent II and III, and preventing it is a primary focus. Segmental arteries between T8 and L2 have been considered to play an important role in spinal cord protection and majority of institutes routinely incorporate large intercostal arteries into the surgery to restore spinal cord perfusion^13,30^. However, those arteries are mostly reimplanted as a single patch through side-by-side anastomosis, even when pre-sewn thoracoabdominal aortic aneurysm graft is used, which also increases the risk of future patch aneurysm, particularly in Marfan patients. To reconstruct a thoracoabdominal aorta using multi-branched aortic graft personalized to reflect individual anatomical variation, we introduce the use of MBT of 3DP for the open surgical repair of thoracoabdominal aortic aneurysm with reasonable operative outcomes. Furthermore, recently, we incorporated the GBT into the MBT to complement it and improve the procedural efficiency and accuracy. The aim of this retrospective study is to quantitatively evaluate these techniques.

In addition to the complexity of this high-risk open repair, it is difficult to ensure that vital branch arteries are in the proper positions when constructing patient-specific grafts. To overcome these challenges, Park et al. developed the octopod technique, an eight-branched aortic graft for reconstruction based on the IBT^14^. However, because the graft was reconstructed by measuring the diagonal line, height, and angle based on CT images under anaesthetic induction in the operating room, it is difficult to understand the individual characteristics of patients in detail within a limited time, which affects the accuracy. For example, the second patient, who had Crawford extent II thoracoabdominal aortic aneurysm with severe scoliosis, had an extremely tortuous main aorta, affecting the visceral and segmental arteries, unlike the general anatomical structure^4^. Because the shape of the graft to be replaced should be connected to the aorta along the tortuous spine, the graft marked using the conventional IBT, calculated by slice thickness and number, may differ from the actual visceral arteries and segmental arteries positions. However, the MBT and GBT using graft reconstruction guides consisting of visualization model and marking guide efficiently reduce the graft marking time and solve anatomical hurdles. In our previous study, Rhee et al.^31^ mentions that the experience on 3DP guidance in open surgical repair of thoracoabdominal aortic dissection and aneurysm have yet been limited in a small number of cases to allow adequate comparisons for its time efficiency in the real clinical practice. Nevertheless, of the 20 patients, 9 patients with 3DP had a shorter duration of cardiopulmonary bypass time than 11 patients, undergoing surgery without 3DP (median, 170 min vs 181 min) despite they had a greater number of segmental arteries revascularized (median, 2 vs. 3), which is mostly likely to require longer cardiopulmonary bypass times. Although the sample size is small, 3DP are assumed that the it is likely to give chance to more complete procedures with greater number of segmental artery anastomoses to prevent paraplegia and to decrease cardiopulmonary bypass time.

In this study, using three techniques, 135 mimic grafts (15 patients, three researchers, and three techniques) ranging from aortic arch to iliac artery were evaluated in experiments. The grafts are a very realistic mimic of standard cylindrical model grafts they were used to bypass the expensive graft cost. The DGM, conventional IBT, MBT, and GBT were evaluated and compared based on graft marking accuracy and time requirements. The errors of the experiments can be divided into marking error with medical images, 3DP errors, and human errors^10,32,33^. The marking errors of the conventional IBT were affected by the subjective measurement in CT images. The direction of the segmental arteries was measured from the centre points of the aorta and the segmental arteries, with the horizontal line. However, for the aneurysm patients, it manifested as elliptical, rather than circular, in the axial view, and the errors could be attributable to the incorrectness of the centre point of the aorta and the subjectivity of the centre point of the aorta and blood vessel (Supplementary Fig. S3)^32,33^. The differences of MBT and GBT arose from 3DP technologies errors, including machine and 3D printed model and guide errors. The machine error is triggered by environmental factors, such as temperature, humidity, and vibration, and the materials, resolution, and usage duration of the 3D printers. The 3D printed model and guide errors depend on the size, shape, printing direction, and printing angle of the guides and post-processing, including support removal, ultraviolet polymerization, and surface smoothing^10,32^. In addition, autoclave and steam sterilization with high temperatures can deform or distort the graft reconstruction guide^34^. To minimize such problems, we opted for ethylene oxide gas sterilization at a relatively low temperature. Three techniques were prone to human errors; errors also occurred when defined landmarks—diagonal line, height, and angle—are measured using a digital calliper and digital angle ruler in the experiments by three researchers. Even if only two landmarks were selected among the diagonal line, height, and angle, the third could be derived; however, the landmarks were measured directly without using a mathematical formula considering measurement error.

In the Bland–Altman analysis, the diagonal line, height, and angles in the conventional IBT at − 45.96 to 33.83 mm, − 46.70 to 33.34 mm, and − 39.05° to 13.05°, respectively, exhibited wider ranges of differences, compared to the MBT and GBT; the differences among the diagonal line and height tended to fall in the negative or positive region as the distance between the segmental artery and celiac artery increased, and the differences between the angles tended to be mostly distributed in the negative region from zero. Outliers tended to appear in the IBT as the distances from the celiac artery from the segmental arteries increased in the diagonal line and height, and occurred in patient with tortuous and swelling aneurysm aorta or scoliosis in the angle (Fig. 5a–c). In the MBT, the difference ranges for diagonal line, height, and angle were − 21.92 to 20.39 mm, − 25.54 to 18.73 mm, and − 39.97° to 23.04°, respectively, and − 21.22 to 19.29 mm, − 12.56 to 14.34 mm, and − 40.61° to 15.31°, respectively, in the GBT (Table 2). The diagonal line and height in the MBT and GBT were distributed in the region of the negative difference, as the measured distance was smaller, indicating a positive difference as the measured distance increased. The bias of the diagonal line and angles was greater in the GBT and the height was greater in the MBT (Fig. 5d–i). In the outliers of the MBT and GBT, the impact on the morphology of the aorta was greater than the distance of the segmental arteries. The MBT and GBT reduced the graft marking time significantly, and the effect was proportional to the number of segmental arteries (Supplementary Fig. S4). For example, the graft marking time for the first patient with the least number of segmental arteries was 10.5, 0.5, and 2.8 min with the conventional IBT, MBT, and GBT, respectively. For the fourteenth patient with 13 segmental arteries, graft reconstruction time was 36.5, 1.4, and 4.4 min with the IBT, MBT, and GBT, respectively. The average time requirement for the MBT and GBT reduced by 17.4 and 15.5 min, respectively, showing the effect reduced by more than six times compared to IBT. In addition, although, based on the marking time, the MBT was more efficient than the GBT, the GBT had superior accuracy (Table 2 and Supplementary Table S2).

The correlation coefficient (r_14_) for the diagonal line and height between the DGM and GBT was observed to be stronger than that of the IBT (r_12_) and MBT (r_13_), whereas a similar level of correlation was confirmed for the angles. Thus, it was implied that the GBT was the most similar to DGM (Table 3). In addition, the ICC was examined, to confirm the human error and the reproducibility of graft reconstruction among three researchers in conventional IBT and new techniques. All the techniques showed high agreement, with 0.995 for the GBT and 0.986 for the IBT and MBT.

For the conventional IBT (without 3DP), and the MBT and GBT (with 3DP), questionnaires were administered to three surgeons with experience in three techniques. Considering the survey score, MBT and GBT with the graft reconstruction guides could provide significantly better help to surgeons to the conventional IBT.

There are several limitations to this study. First, the procedures, from data acquisition to 3DP, lasted over a week, which made it difficult to apply the reconstruction techniques using patient-specific 3D printed graft reconstruction guides to open repair in patients with acute aortic rupture. Seconds, depending on the quality of the CT images, low-resolution images or slice thickness exceeding 3 mm made it difficult to identify the segmental arteries. Finally, improvements in efficacy and efficiency in the real clinical setting are the final purposes of using 3DP in the open repair of TAAA, however, the best available data on this issue have been limited by a small sized sample to allow adequate comparisons of hard clinical endpoints such as mortality, surgical bleeding, neurologic injuries and end-organ failures depending on the use of 3DP. In further studies, to reduce the graft reconstruction time, not only should the position of the segmental arteries on the graft be marked, functions, including the suturing and bonding process and the application for direct bioprinting process, should be developed. In addition, comparisons in clinical outcomes through larger cohort as the next step research are required to validate the usefulness of 3DP in open TAAA repair.

## Conclusion

In this study, two types of grafts were developed for patient-specific graft reconstruction. Both methods provided a useful individualised approach to the anatomically challenging open repair. The MBT and GBT effectively reduced the graft reconstruction time and improved the accuracy, compared to the IBT.

## Supplementary Information

Supplementary Table S1.

Supplementary Table S2.

Supplementary Figure S3.

Supplementary Figure S4.
