# Supplementary material for: Utilizing patient-specific 3D printed guides for graft reconstruction in thoracoabdominal aortic repair
Source: Sci Rep. 2021 Sep 9;11:18027. doi: 10.1038/s41598-021-97541-8 (PMC8429675; doi:10.1038/s41598-021-97541-8)
Supplement: Supplementary file 1 — Supplementary Table S1. [file 41598_2021_97541_MOESM1_ESM.pdf]

Supplementary Table S1. Survey form for evaluating graft reconstruction guide for surgery using 3 techniques (IBT, MBT and GBT) with 3 surgeons.

**Graft reconstruction with 3D printing guides (MBT and GBT)**	Score
➢ Understanding the anatomic structure with 3D printing	
1. The 3D printed models and guides are helpful understanding the patient's anatomy before surgery.	5.00 ± 0.00
2. The 3D printed models and guides are helpful in identifying blood vessels.	4.33 ± 0.58
3. The 3D printed model and guide help to determine the extent of aortic dissection.	3.00 ± 1.00
4. The 3D printed models and guides help me plan our surgery.	5.00 ± 0.00
➢ Usefulness for guide-based technique with 3D printing	
5. The 3D printed model and guide are designed for the purpose	4.67 ± 0.58
6. 3D printed models and guides help graft reconstruction.	5.00 ± 0.00
7. The use of 3D printed models and guides is more efficient than conventional graft reconstruction methods.	5.00 ± 0.00
➢ Satisfaction for guide-based technique with 3D printing	
8. I have a positive attitude about the 3D printed models and guides.	5.00 ± 0.00
9. The 3D printed models and guides were fabricated to be convenient to use.	4.67 ± 0.58
➢ Surgical outcomes with 3D printing	

10. The 3D printed models and guides can efficiently reduce the operation time.	4.67 ± 0.58
11. The 3D printed models and guides help to improve my surgical outcomes.	4.67 ± 0.58
➢ Recommendations for other applications.	
12. The use of 3D printed models and guides will recommend to other surgeons.	4.33 ± 0.58
13. I hope that 3D printed models and guides will be applied in other fields as well.	4.00 ± 0.00
**Graft reconstruction without 3D printing guides (IBT)**	Score
➢ Understanding the anatomic structure without 3D printing	
1. The conventional technique is more helpful in understanding the patient's anatomy prior to surgery than using 3D printed models and guides.	1.67 ± 0.58
2. The conventional technique is more helpful in identifying blood vessels than using 3D printed models and guides.	2.00 ± 1.00
3. The conventional techniques are more helpful in determining the extent of aortic dissection than using 3D printed models and guides.	2.33 ± 1.15
4. The conventional techniques are more helpful in planning surgery than using 3D printed models and guides.	1.67 ± 0.58
➢ Usefulness for image-based technique without 3D printing	
5. Because 3D printed models and guides are not designed for the purpose, the conventional technique is more useful.	1.00 ± 0.00
6. The conventional technique is more helpful in reconstructing the graft than using 3D printed models and guides.	1.67 ± 0.58

7. The use of 3D printed models and guides is more efficient than conventional graft reconstruction methods.	1.00 ± 0.00
➢ Satisfaction for image-based technique without 3D printing	
8. I have a positive attitude about the conventional technique than using 3D printed models and guides.	1.67 ± 0.58
9. Because 3D printed models and guides are inconvenient to use, the conventional technique is preferred.	1.67 ± 0.58
➢ Surgical outcomes without 3D printing	
10. The conventional techniques can further reduce surgical time than using 3D printed models and guides.	1.67 ± 0.58
11. The conventional technique helps to improve the surgical outcome than using 3D printed models and guides.	1.67 ± 0.58
➢ Recommendations for other applications	
12. The use of 3D printed models and guides will be not recommended to other surgeons.	1.67 ± 0.58
13. 3D printed models and guides are unlikely to be helpful if applied in other fields.	1.67 ± 0.58
