# Supplementary material for: Utilizing patient-specific 3D printed guides for graft reconstruction in thoracoabdominal aortic repair
Source: Sci Rep. 2021 Sep 9;11:18027. doi: 10.1038/s41598-021-97541-8 (PMC8429675; doi:10.1038/s41598-021-97541-8)
Supplement: Supplementary file 2 — Supplementary Table S2. [file 41598_2021_97541_MOESM2_ESM.pdf]

Supplementary Table S2. Marking time requirements spent by 3 researchers on 15 patients using 3 techniques (IBT, MBT and GBT), IBT, image-based technique; GBT, guide-based technique.

Patients	Techniques	Observer 1 (min)	Observer 2 (min)	Observer 3 (min)	Mean ± SD
1	IBT	10.48	10.45	10.40	10.44 ± 0.04
	MBT	0.60	0.51	0.37	0.49 ± 0.11
	GBT	3.03	3.45	3.50	3.33 ± 0.25
2	IBT	13.50	11.53	9.58	11.53 ± 1.96
	MBT	0.93	0.41	0.55	0.63 ± 0.26
	GBT	2.38	3.27	3.43	3.03 ± 0.56
3	IBT	24.60	33.92	25.27	27.93 ± 5.19
	MBT	0.94	0.51	1.87	1.11 ± 0.69
	GBT	3.18	3.12	2.27	2.86 ± 0.50
4	IBT	11.48	10.93	13.87	12.09 ± 1.56
	MBT	1.17	0.26	0.63	0.69 ± 0.45
	GBT	3.67	3.45	3.18	3.43 ± 0.24
5	IBT	10.28	13.95	10.20	11.47 ± 2.14
	MBT	0.87	0.32	1.02	0.74 ± 0.36
	GBT	2.23	1.22	2.82	2.09 ± 0.80
6	IBT	12.03	9.77	16.00	12.60 ± 3.15
	MBT	0.73	0.17	0.60	0.50 ± 0.29
	GBT	3.53	1.88	2.15	2.52 ± 0.88
7	IBT	14.90	12.22	10.38	12.50 ± 2.27
	MBT	1.10	0.44	0.38	0.64 ± 0.39
	GBT	1.93	2.40	2.12	2.15 ± 0.23
8	IBT	10.70	13.68	13.78	12.72 ± 1.75
	MBT	1.50	0.40	0.52	0.81 ± 0.60
	GBT	2.27	2.57	3.22	2.69 ± 0.48
9	IBT	10.80	9.63	12.57	11.00 ± 1.48
	MBT	0.87	0.55	0.32	0.58 ± 0.27
	GBT	1.80	1.87	1.78	1.82 ± 0.04
10	IBT	23.42	17.90	21.22	20.84 ± 2.77
	MBT	1.79	0.50	0.62	0.97 ± 0.71
	GBT	2.58	1.55	2.18	2.10 ± 0.51
11	IBT	18.62	22.87	15.72	19.07 ± 3.59
	MBT	1.27	0.59	0.62	0.83 ± 0.38
	GBT	3.13	2.57	3.83	3.18 ± 0.63
12	IBT	15.95	13.17	22.58	17.23 ± 4.83
	MBT	1.04	0.62	0.37	0.68 ± 0.33

	GBT	2.05	1.53	2.98	2.19 ± 0.73
13	IBT	28.62	21.55	21.40	23.85 ± 4.12
	MBT	1.21	0.59	1.05	0.95 ± 0.32
	GBT	3.17	3.07	4.13	3.46 ± 0.58
14	IBT	40.27	31.05	38.03	36.45 ± 4.80
	MBT	2.66	0.59	0.92	1.39 ± 1.11
	GBT	4.20	2.45	6.57	4.41 ± 2.06
15	IBT	38.67	31.28	41.00	36.98 ± 5.07
	MBT	1.47	0.70	1.00	1.06 ± 0.38
	GBT	4.97	3.08	6.22	4.76 ± 1.58
