# Supplementary material for: Utilizing patient-specific 3D printed guides for graft reconstruction in thoracoabdominal aortic repair
Source: Sci Rep. 2021 Sep 9;11:18027. doi: 10.1038/s41598-021-97541-8 (PMC8429675; doi:10.1038/s41598-021-97541-8)
Supplement: Supplementary file 3 — Supplementary Figure S3. [file 41598_2021_97541_MOESM3_ESM.pdf]

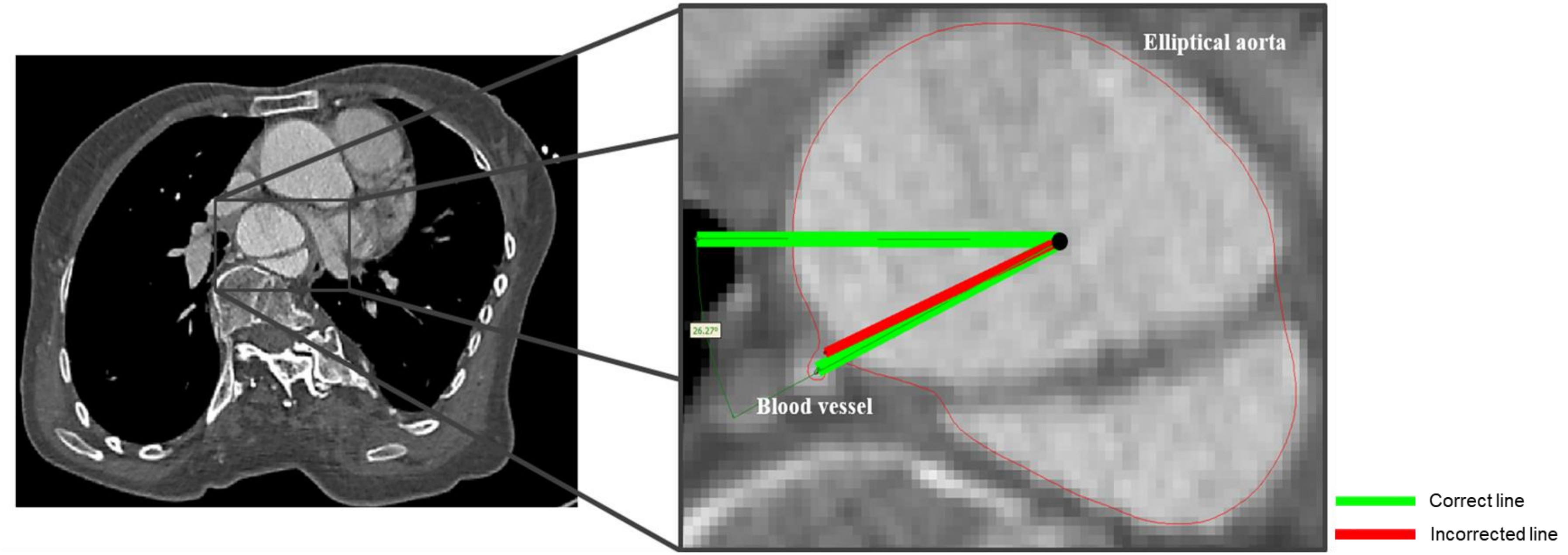

Supplementary Figure S3. Figure that shows the subjective assessment and measurement error of elliptical aortic center and blood vessels in CT images
