# Supplementary material for: Utilizing patient-specific 3D printed guides for graft reconstruction in thoracoabdominal aortic repair
Source: Sci Rep. 2021 Sep 9;11:18027. doi: 10.1038/s41598-021-97541-8 (PMC8429675; doi:10.1038/s41598-021-97541-8)
Supplement: Supplementary file 4 — Supplementary Figure S4. [file 41598_2021_97541_MOESM4_ESM.pdf]

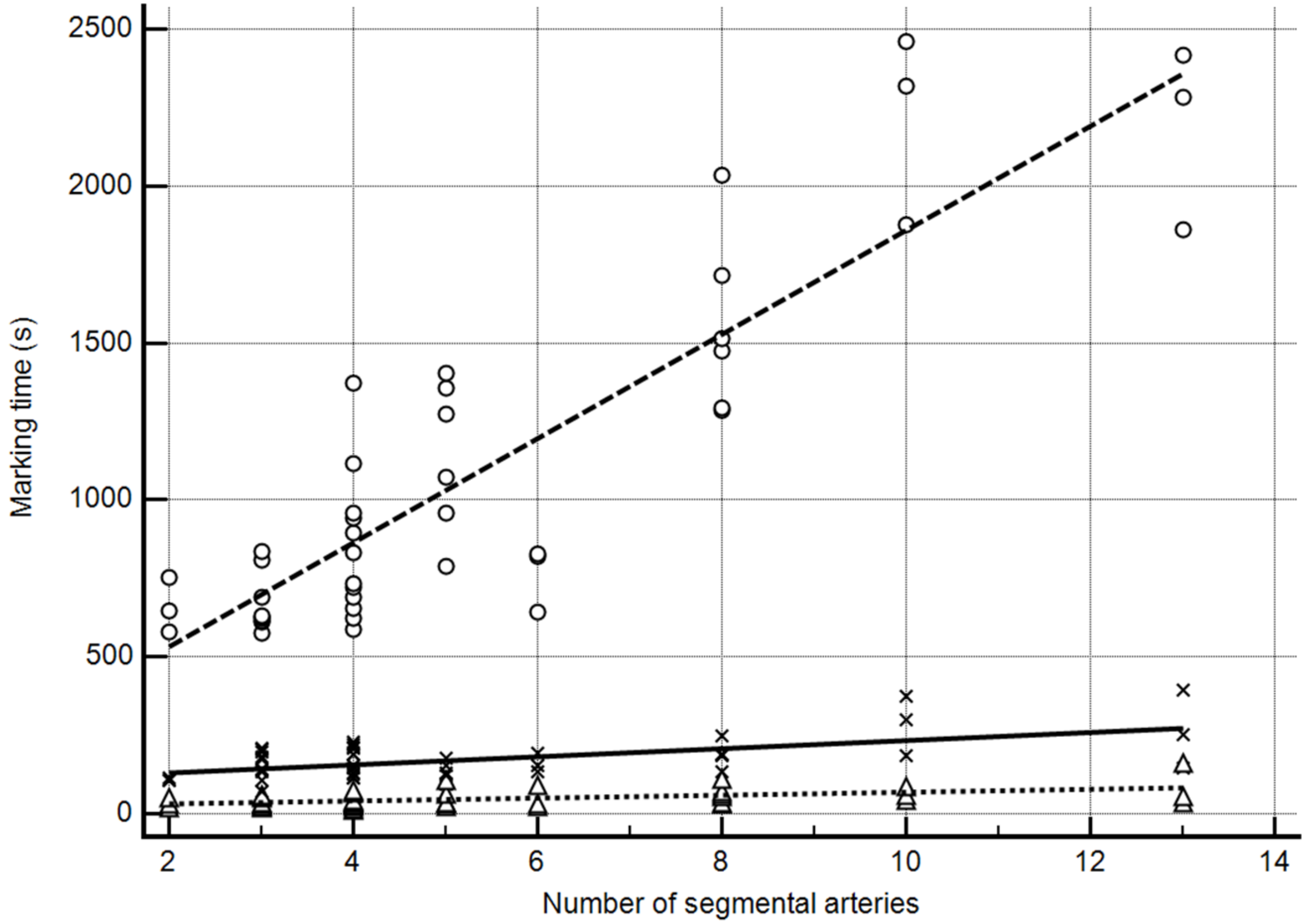

Supplementary Figure S4. Marking time according to the number of segmental arteries for IBT, MBT, and GBT.
